# Antiadherent and Antibiofilm Activity of *Humulus lupulus* L. Derived Products: New Pharmacological Properties

**DOI:** 10.1155/2013/101089

**Published:** 2013-09-23

**Authors:** Marcin Rozalski, Bartlomiej Micota, Beata Sadowska, Anna Stochmal, Dariusz Jedrejek, Marzena Wieckowska-Szakiel, Barbara Rozalska

**Affiliations:** ^1^Department of Haemostatic Disorders, Medical University of Lodz, Zeromskiego 113, 90-549 Lodz, Poland; ^2^Department of Infectious Biology, Faculty of Biology and Environmental Protection, University of Lodz, Banacha 12/16, 90-237 Lodz, Poland; ^3^Department of Biochemistry, Institute of Soil Science and Plant Cultivation, State Research Institute, Czartoryskich 8, 24-100 Pulawy, Poland

## Abstract

New antimicrobial properties of products derived from *Humulus lupulus* L. such as antiadherent and antibiofilm activities were evaluated. The growth of gram-positive but not gram-negative bacteria was inhibited to different extents by these compounds. An extract of hop cones containing 51% xanthohumol was slightly less active against *S. aureus* strains (MIC range 31.2–125.0 **μ**g/mL) than pure xanthohumol (MIC range 15.6–62.5 **μ**g/mL). The spent hop extract, free of xanthohumol, exhibited lower but still relevant activity (MIC range 1-2 mg/mL). There were positive coactions of hop cone, spent hop extracts, and xanthohumol with oxacillin against MSSA and with linezolid against MSSA and MRSA. Plant compounds in the culture medium at sub-MIC concentrations decreased the adhesion of *Staphylococci* to abiotic surfaces, which in turn caused inhibition of biofilm formation. The rate of mature biofilm eradication by these products was significant. The spent hop extract at MIC reduced biofilm viability by 42.8%, the hop cone extract by 74.8%, and pure xanthohumol by 86.5%. When the hop cone extract or xanthohumol concentration was increased, almost complete biofilm eradication was achieved (97–99%). This study reveals the potent antibiofilm activity of hop-derived compounds for the first time.

## 1. Introduction

Hops, the resinous female inflorescences of *Humulus lupulus* L. (*Cannabaceae*) (called hop cones or strobiles), are used primarily in the brewing industry because of their bitter and aromatic properties. However, hop extracts and/or compounds such as polyphenols and acylphloroglucides are also reported to have antioxidant, estrogenic, sedative, and potential cancer-chemopreventive activities. Xanthohumol is the most abundant prenylated flavonoid in fresh hops, with properties overlapping those mentioned above [1–4]. Most interesting for our research is the antimicrobial activity of xanthohumol and other hop extract compounds, which could find new applications beyond the brewing industry as natural antimicrobial therapeutic substances. Research on the use of hop products to combat human pathogens has been conducted all over the world, and knowledge in this area from *in vitro* studies is already quite extensive, though further studies are still required [5–9]. Here, we wish to propose an investigation into an entirely new potential use of hop products, as antibiofilm compounds and enhancers of antibiotic action. Biofilm formation has substantial implications for a variety of industries such as oil drilling, paper production, and food processing. It is also well known that bacterial and fungal pathogens that form biofilms are responsible for serious infections, which are usually very difficult to treat. This is due to the high resistance of biofilms (100–1000 times higher than for a planktonic culture) to antibiotics, antiseptics, disinfectants, and host defense mechanisms [10]. This justifies the search for new therapeutic options, and plant-derived products are in the spotlight as promising sources or templates for new drugs [11, 12]. 

The aim of our study was to establish the antibacterial activities of a purified extract from hop cones containing 51% xanthohumol and a spent hop extract depleted of xanthohumol and to compare them with commercially available xanthohumol. The first stage of the study comprised MIC/MBC evaluation of the above products and their synergy with antibiotics. We then examined the effects of these phytochemicals against staphylococcal biofilms, which have not been evaluated previously. Owing to the high resistance of biofilms, the ideal way of avoiding their engagement in *in vivo* pathogenesis or in other biofouling processes in the environment and industry would be to prevent their development. Therefore, one of our objectives was to evaluate the adhesion of *S. aureus* strains to glass/plastic surfaces and biofilm formation when hop constituents were continually present. We also assessed the capacity of these phytocompounds to eradicate an already-established biofilm. 

## 2. Materials and Methods

### 2.1. Extraction, Isolation, and Chemical Analysis of Phytocompounds

 Hop cones var. Marynka were grown at the experimental farm of the Institute of Soil Science and Plant Cultivation, State Research Institute of Pulawy, Poland. Plant material was collected during the 2010 season. Hop cones were dried at 55°C and kept in a cooler (4°C) pending extraction. Hop cones (100 g) were powdered and extracted with 2 L of 70% ethanol (EtOH) by boiling for 60 min. The extract was filtered and evaporated at 40°C to remove the organic phase, and then the crude extract was left at room temperature for 2 h until the sediment was separated from the liquid phase. This process was accelerated by centrifuging the extract (15 min, 5.000 ×g). The precipitate, which contained most of the xanthohumol, was freeze-dried, suspended in 30% EtOH, and applied to a C18 preparative column (45 × 160 mm, 40–63 *μ*m LiChroprep, Merck) previously preconditioned with 30% EtOH in 1% acetic acid (AcOH). The column was washed with linearly increasing concentrations of EtOH (from 30% to 100%) in 1% AcOH. Ten mL fractions were collected and monitored by HPLC. Fractions containing xanthohumol were combined and freeze-dried. After this stage, the quantity of xanthohumol in the extract was measured by HPLC. The final xanthohumol content amounted to 51% of the dry matter, as determined by the HPLC system (Waters, USA), which comprised a Waters 600 controller, a 616 pump with an in-line degasser AF, and a model 717 plus autosampler, as described previously [2]. A calibration curve was prepared for xanthohumol (Sigma, USA) at *λ* = 370 nm.

Spent hops, after extraction of the hop cones by supercritical CO_2_, were supplied by the Fertilizer Research Institute of Pulawy, Poland. A dried sample was ground to fine powder and suspended in acetone-water (70 : 30, v/v) at a solid to liquid ratio 1 : 10, mixed at room temperature for 30 min, and then centrifuged for 15 min (4.000 rpm). The pellet was reextracted three times with 70% aqueous acetone at room temperature, with stirring, and then the extracts were filtered and concentrated to remove the organic solvent. Lipophilic compounds were removed from the extract using chloroform and dichloromethane. The defatted aqueous extract was then concentrated to remove any residual solvent, concentrated under vacuum, and freeze-dried. Before analysis, the dried extract was reconstituted at 2 mg/mL in 10% aqueous dimethyl sulfoxide (DMSO). Total phenols, flavanols, and proanthocyanidins were determined using the methods described, respectively, by Bordonaba and Terry [13], Swain and Hillis [14], and Rösch et al. [15]. Polyphenols in the extracts were identified using an Acquity Ultra Performance LCTM system (UPLCTM) with a binary solvent manager (Waters Co., Milford, USA) and a Micromass Q-TOF Micromass spectrometer (Waters, Manchester, UK), equipped with an electrospray ionization (ESI) source operating in negative and positive modes. The individual components were characterized via their retention times and accurate molecular masses. The data obtained from UPLC/MS were analyzed with MassLynx 4.0 ChromaLynxTM Application Manager software. 

### 2.2. Evaluation of Minimum Inhibitory Concentration (MIC) and Minimum Bactericidal Concentration (MBC) of the Phytocompounds

 The reference strains of *Staphylococcus aureus *ATCC 29213, *Enterococcus faecalis* ATCC 29212, *Escherichia coli* NCTC 8196, *Pseudomonas aeruginosa* NCTC 6749, and the clinical *S. aureus *strains A7 and D5 were used. Bacteria were grown for 24 h at 37°C on Müeller-Hinton Agar—MHA (BTL, Poland), and microbial suspensions (5 × 10^5^ CFU/mL) were prepared in Müeller-Hinton Broth—MHB (BTL, Poland). MIC values were determined by a microdilution broth assay according to Clinical and Laboratory Standards Institute (CLSI) recommendations [16, 17]. Stock solutions of hop cone, spent hop extracts, and xanthohumol (Sigma, USA) were prepared in 50% spent hop extract or 100% DMSO. The concentration ranges of the compounds used in the tests (using a twofold dilution system) were 0.0039–0.5 mg/mL for XH and 0.0078–2.0 mg/mL for the extracts. These ranges were based on general assumptions underpinning research on natural products for medical use, which set the upper limit for biostatic/biocidal concentration at about 1 mg/mL for complex preparations and 0.1 mg/mL for pure chemical compounds.

Bacterial suspensions (100 *μ*L) were mixed 1 : 1 with the serial dilutions of the phytocompounds under test. Microplate wells containing no extract but inoculated with test strains were used as positive controls. Negative control wells consisted of the serial dilution of the phytocompound only. The final highest DMSO concentration was 1.25%, which did not affect bacterial growth. Plates were incubated at 37°C for 18 h, and the highest dilution showing no turbidity was recorded as the MIC. Since the color of the extracts at higher concentrations made turbidimetry difficult, bacterial growth on MHA (10 *μ*L from each well after vigorous stirring; linear culture incubated for the subsequent 18 h at 37°C) was tested concurrently. The concentrations of the compounds bactericidal to ≥99.9% of the inoculum (MBC) were determined using the same method of solid culture (starting from four wells below the suspected MIC value). In each case, experiments were carried out in quadruplicate on two different experiments. In order to test whether the compounds induced cell aggregation, 25 *μ*L of bacterial suspension, treated as described above, was placed on a glass microscope slide and gently smeared. After air drying, heat fixation, and gram staining, the slides were examined by light microscopy. The size and quantity of clusters were compared to the controls (nontreated bacteria) according to the score established by Cushnie et al. [18].

### 2.3. Determination of Antibiotic Synergy with Hop-Derived Products, Assessed by the E-Test Strip/Agar Dilution Method

 Prepared inocula of *S. aureus* ATCC 29213 (MSSA) and the clinical *S. aureus* MRSA strains D5 and A7 (1 × 10^8^ CFU/mL) were spread with a sterile cotton swab on (a) control MHA or (b) MHA containing hop cone or spent hop extract or xanthohumol (at a final concentration of 1/2 MIC or 1/4 MIC). At the first stage a standard disk-diffusion test was performed according to CLSI recommendations [16, 17], using the following antibiotic set: oxacillin (1 *μ*g/disc), cefoxitin (30 *μ*g/disc), clindamycin (2 *μ*g/disc), vancomycin (30 *μ*g/disc), and erythromycin (15 *μ*g/disc) (Mast Diagnostics, UK). Antibiotic gradient strips (E-test, BioMerieux, France) containing oxacillin, vancomycin, or linezolid (concentration range 0.016–256 mg/L) were then used; the MHA plates with the overlayered strips were incubated at 37°C for 24 h, and the growth inhibition zones were measured. Differences in MIC values between the control and test plates were recorded (end points were determined according to the manufacturer's instructions).

### 2.4. *S. aureus* Adhesion, Biofilm Formation, and Biofilm Eradication under the Influence of Hop Constituents

 A suspension of *S. aureus* ATCC 29213 (OD = 0.6, which corresponded to a density about 1 × 10^7^ CFU/mL) prepared from a fresh overnight culture in tryptic soy broth (TSB, Difco, USA) supplemented with 0.25% glucose (TSB/Glc) was added (100 *μ*L) to the wells of a 96-well tissue culture polystyrene microplate (Nunc, Denmark). To estimate bacterial adhesion, standardized glass carriers (5 mm diameter; Thermo Scientific, Germany) were put into the wells followed by 100 *μ*L of the phytochemicals under test at final concentrations of 1/2 MIC, 1/4 MIC, and 1/8 MIC (in quadruplicate for each concentration). Glass carriers placed in bacterial culture alone (without hop constituents) were used as positive controls. Negative control wells consisted of glass carriers in phytocompounds (1/2 MIC) and TSB/Glc only. After 2 h incubation at 37°C, the glass carriers were removed, vortexed (3 min), serially diluted (10-fold dilution series in 0.85% NaCl), and cultured on MHA plates (100 *μ*L/plate; 24 h, 37°C). The percentage of bacterial adhesion in the presence of phytocompounds was compared to that in the control culture on the basis of CFU counts. To evaluate biofilm formation, a LIVE/DEAD BacLight Bacterial Viability kit (Molecular Probes, USA) was used as recommended by the manufacturer. Bacterial suspensions (OD = 0.6) were cultured on microplates (100 *μ*L/well) at 37°C in the absence (control) or constant presence of the phytocompounds (1 : 1 ratio with bacteria) at their final 1/2, 1/4, and 1/8 MICs. After 24 h incubation, free-floating bacterial cells were gently removed from the wells, and the remaining biofilm was stained with Syto9 and propidium iodide (PI) (15 min in the dark). Finally, the dyes were replaced with water (200 *μ*L/well) and the fluorescence of the wells (at 485ex/535em nm for green Syto9 and at 485ex/620em nm for red PI) was measured. The results are presented as percentage biofilm biomass calculated from the mean fluorescence values ±S.D. of the control (considered as 100%) and test wells. Another set of plates was designed to investigate the influence of the MIC or 2xMIC of each phytocompound on preformed *S. aureus* ATCC 29213 biofilms (24 h old), starting from the same bacterial suspension. After a subsequent 24 h incubation of the staphylococcal biofilm at 37°C with or without (control) the hop constituents under test, the degree of biofilm survival (%) was assessed as described above using staining with the LIVE/DEAD Bacterial Viability assay.

### 2.5. Statistical Analysis

 If necessary, differences in parameters were tested for significance using the Mann-Whitney *U* test and the program Statistica 5.0 [Stat Soft Inc.].

## 3. Results and Discussion

Separation of the precipitate from the ethanolic extract of the powdered hops on a preparative C18 column resulted in a fraction that contained 51% dry weight xanthohumol. The HPLC profile showed the presence of one predominant compound. The other small peaks seen in the profile belonged to isoxanthohumol and alpha-acids, but the peak area indicated that they did not exceed 2% of the sample dry mass, as described previously [2]. 

The total phenol content of the spent hop extract was about 24%; half the phenols were flavanols. A total of 10 flavan-3-ols were identified using ultraperformance liquid chromatography-mass spectrometry. Two monomers ((+)-catechin and (−)-epicatechin), four dimers, and four trimers were detected. The spent hop extract also contained four hydroxycinnamates: neochlorogenic acid, chlorogenic acid, cryptochlorogenic acid, and feruloylquinic acid. Flavonols were represented by quercetin and kaempferol derivatives. This extract did not contain xanthohumol (achieved by additional purification steps), since our research was intentionally directed towards the utilization of the spent hops after extraction of this compound from the waste.

The hop cone extract, spent hop extract, and (for comparison) pure xanthohumol were assessed for antimicrobial activity *in vitro*. Initially, the antibacterial effect of these products was evaluated against a panel of reference strains. The growth of gram-negative bacteria (*Escherichia coli and Pseudomonas aeruginosa*) was not inhibited by any of the investigated compounds in relevant concentrations (the MICs exceeded their highest used concentrations, data not shown). In contrast, the hop cone extract was a potent antagonist of the gram-positive *Staphylococcus aureus* ATCC 29213 (MIC 31.3 *μ*g/mL) and *Enterococcus faecalis* ATCC 29212 (MIC 62.5 *μ*g/mL), exhibiting half the efficacy of pure xanthohumol (Table 1). The spent hop extract (free of xanthohumol but containing significant amounts of various quercetin and kaempferol derivatives, catechin, and epicatechin), not previously studied in this regard, exhibited much lower but still significant activity against gram-positives (MICs range 1-2 mg/mL). For comparison, as presented in our previous work, the MICs of the reference flavonoids quercetin and naringin were >300 *μ*g/mL and those of thymol and kaempferol were, respectively, 18.75 *μ*g/mL and 62.5 *μ*g/mL [19]. A standard set of microorganisms used to assess antimicrobial activity of various preparations covers a broad panel of microbes, including both Gram-positive (represented by *Staphylococci* and enterococci) and gram-negative (fermenting and nonfermenting bacilli) bacteria. The reasons for their behavior towards biologically active substances can be ascribed in their structure or metabolism. Our results on antimicrobial activity of studied phytocompounds divided the group of test microorganisms into two subgroups, susceptible and resistant, which correspond to the division on gram-positive and gram-negative bacteria. This demonstrates the importance of structure of cell wall/membrane and its permeability rather than metabolic activity of these microorganisms. It is not without significance the fact that, as was described by Sakai et al. [20], xanthohumol (an inhibitor of diacylglycerol acyltransferase) and natural products which contain this compound (hop extract) belong to the group of products which are potent inhibitors of lipid metabolism. This can significantly affect the composition and stability of microbial cell wall/membrane. But our experimental results do not allow us to explain the mechanisms of biological activity of tested hop extract, spent hops extract, and xanthohumol accurately, and this requires further detailed studies.

The obtained results encouraged us to undertake further studies on the activities of *H. lupulus-*derived products against multidrug-resistant *S. aureus* strains. For the experiment, we selected two clinical isolates—members of an important group of “alert” human pathogens—MRSA (methicillin-resistant *S. aureus*) A7 and D5. The hop-derived components demonstrated potent activity, but their MICs against the D5 strain were higher than those reported earlier for *S. aureus* ATCC 29213 (MSSA) (Table 1). Following MIC evaluation, inhibition of bacterial growth on solid media revealed ≥99.9% reduction of the original inoculum by hop cone and spent hope extracts or xanthohumol. The results proved that the phytocompounds tested exhibited concentration-dependent bactericidal effects (MBC) (Table 1). In order to test whether the compounds induced cell aggregation, which could influence CFU counts during MBC testing, samples of bacteria incubated with the phytocompounds were compared under light microscopy with controls (untreated bacteria). Most of the bacteria incubated with hop cone and spent hop extracts (at MIC) formed similar numbers of pairs and small clusters as the control, while bacteria treated with xanthohumol were found mainly in small and large aggregates (data not shown). This effect disappeared when the concentration of xanthohumol was reduced to 1/2 MIC. Therefore, half MIC and two lower concentrations (1/4 and 1/8 MIC) were used in experiments on the influence of phytochemicals on adhesion, biofilm formation, and synergy with antibiotics. As proposed by Cushnie et al. [18] and Cushnie and Lamb [21], aggregation of bacterial cells should always be taken into account when interpreting data from assays with natural flavonoids and flavonoid-rich phytochemical preparations. It is well known that the hop extraction method used determines the composition of the products and their biological activity [22–25]. Papers describing the antibacterial actions of different compounds in hops mainly are concerned with the activities of the bitter acids humulone and lupulone and of the flavonoid xanthohumol [9, 26–29]. Xanthohumol has also been reported as the main component with anti-infective effects against viruses and fungi [6]. 

**Figure 1:**
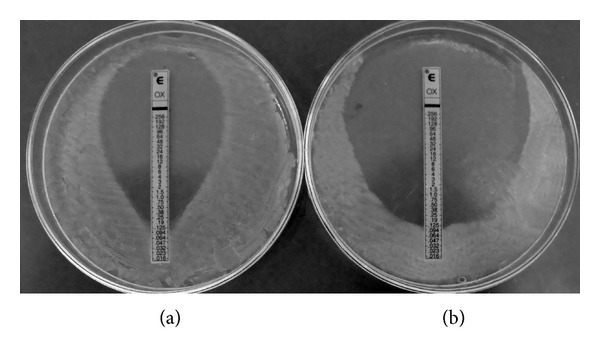
Synergistic effect of oxacillin and xanthohumol against *S. aureus* ATCC 29213 evaluated by E-test strip/agar dilution method. (a) The control plate (MHA); (b) the tested plate (MHA with xanthohumol at final dilution equal to 1/4 MIC).

As pathogens become more and more resistant to available antibiotics, posing a significant medical problem, the need for alternative treatments becomes greater. Several studies have suggested that combining plant- or animal-derived natural compounds with antibiotics is a new strategy for developing therapies against infections [12, 21, 30]. Here, we report that the antimicrobial activities of commercial antibiotics are enhanced by adding hop compounds, forming a potent inhibitor of growth of *S. aureus*. MIC values were decreased by the coactions of hop products with oxacillin (*β*-lactam) and linezolid (oxazolidinone) but not vancomycin (glycopeptide) (Table 2). When hop cone or spent hop extracts or xanthohumol was incorporated into the agar medium at 1/2 MIC or 1/4 MIC, the MIC of oxacillin against MSSA *S. aureus* ATCC 29213 was reduced. The example is shown on Figure 1. Since hop products have previously been demonstrated to affect cell wall and membrane integrity, it is possible that they facilitate antibiotic penetration. This could explain why they potentiate the action of oxacillin, which by binding to specific penicillin-binding proteins (PBP) inside the bacterial cell wall inhibits cell wall synthesis. Unfortunately, the sensitivity of MRSA strains was not increased by hop derivatives. Methicillin resistance in *S. aureus* is primarily mediated by the mecA gene, which encodes the modified PBP 2a. This protein is also located in the bacterial cell wall and has a lower binding affinity for *β*-lactams. Although all cells in a population of *S. aureus* can carry the *mecA* gene, often only a few of them express it. Thus, both resistant and susceptible bacteria can exist in the same culture [31]. Our results confirm this phenomenon. Although the MIC of oxacillin did not decrease when the whole population of MRSA was grown in the presence of phytochemicals, growth was weakened. As mentioned, extracts of hop cones, spent hops and xanthohumol did not increase the sensitivity of the reference or clinical strains to vancomycin. However, there was increased sensitivity to linezolid, a bacteriostatic antibiotic inhibiting the initiation of protein synthesis. It is effective against infections caused by various gram-positive pathogens, including multidrug resistant enterococci and MRSA. However, starting from 2008, cases of isolation of resistant strains have been recorded, luckily still with low incidence [32]. The strengthening of its action that we were able to achieve is interesting and suggests that hop products probably facilitate the penetration of this antibiotic into the bacterial cell. 

**Figure 2:**
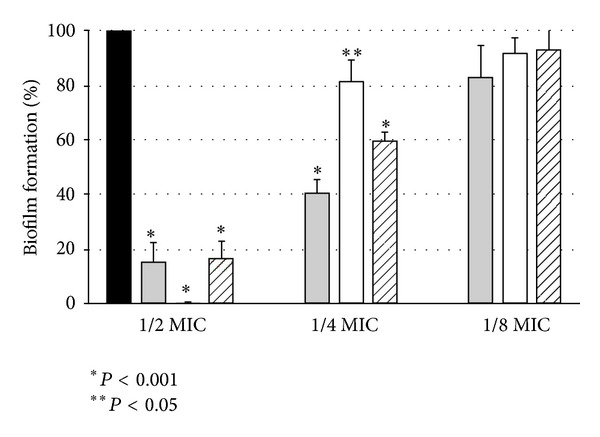
Antibiofilm activity of *Humulus lupulus*-derived extracts and xanthohumol against *S. aureus* ATCC 29213. Bacteria were cultured for 24 h in absence or constant presence of the phytocompounds used at their 1/2, 1/4, and 1/8 MICs. Biofilm formation was assessed using a LIVE/DEAD BacLight kit. Results are presented as the percentage of the biomass viability, compared to the control. All presented results are mean from 2 independent experiments performed in quadruplicate ± S.D. Black bars: control; grey bars: spent hops; open bars: hop cones; striped bars: xanthohumol.

 Why did we use *Staphylococcus aureus* as the model organism in our study? It is obvious that it would be also interesting to test susceptibility of enterococci to products derived from hops. These bacteria, like *Staphylococci*, constitute a serious epidemiological risk since they are well equipped with a variety of natural antibiotic resistance; they are also capable of acquiring new resistance genes and/or mutations. However, our research interest has focused the attention on the *S. aureus* behavior. These are the reasons: first, because these bacteria produce a large number of virulence factors that are important for pathogenesis; secondly, because they are on the list of alarm multiresistant pathogens; finally, because biofilm formation by them is a major medical problem. Since bacteria in biofilms are extremely resistant to antimicrobial agents, biofilm-associated infections are very difficult to treat, especially when the causative organism is multidrug resistant [10]. Thus, another question we asked was whether the hop-derived compounds can be considered effective in antibiofilm therapy. We demonstrated that they were effective (at 1/4 or 1/2 MIC) against staphylococcal adhesion evaluated after 2 h (inhibition range 50–90%) and biofilm formation evaluated after 24 h of coincubation (Figure 2). Moreover, these extracts or pure xanthohumol applied to already-formed biofilms at the relatively low concentrations of MIC or 2xMIC reduced the viability of the mature biofilm significantly. The most potent in this respect was xanthohumol, which reduced the biofilm by 86.5 ± 1.5% (at MIC), whereas the spent hop extract caused 42.8 ± 16.3% and the hop cone extract 74.9 ± 6.7% biofilm eradication at MIC. Given the known high resistance of biofilm populations, our observation should be considered promising, since even partial destruction of a biofilm by antibiotics/antiseptics is encouraging. 

However, our experimental results to date do not allow the mechanisms of biological activity of the tested hop extract, spent hop extract, and xanthohumol to be explained adequately. It can be supposed that their effective penetration across the cell wall and/or membrane damage is the most important property. They could also influence bacterial cell surface hydrophobicity and, depending on sortase activity, the assembly of adhesins in the cell wall [33]. Thus, our plant-derived compounds could interfere with the adhesion step essential for successful biofilm development. Their observed effects suggest that they easily penetrate biological membranes, probably without the help of active transport mechanisms [21], but this possibility needs further research. 

## 4. Conclusions 

In summary, the present study has revealed the potent antibiofilm activity of hop-derived compounds for the first time. This is interesting, particularly with regard to the action of the spent hop extract, which is a quantitatively significant waste from the brewing industry. Therefore, this observation has the potential for practical application, since spent hop extract containing no xanthohumol is still a good source of substances with antimicrobial and antibiofilm activities. Although the mechanisms of biological activity of the phytocompounds tested are not clear (we can only discuss possibilities, as above), our results suggest that hop-derived constituents can be extended beyond the beer industry to prospective medical applications.

## Figures and Tables

**Table 1:** Minimum inhibitory and biocidal concentration (MIC/MBC) of hop-derived compounds against selected *S. aureus* strains, determined by a microdilution broth assay accompanied by assessment of bacterial growth on solid media.

[mg/mL]	Spent hops extract		Hop cones extract		Xanthohumol	
	MIC	MBC	MIC	MBC	MIC	MBC
*S. aureus* 29213	2	>2	0.031	0.065	0.015	>0.5
*S. aureus* D5	1	2	0.125	0.5	0.125	0.5
*S. aureus* A7	2	2	0.031	0.031	0.125/0.062	0.25
*E. faecalis* 29212	>2	>2	0.062	1	0.062	>0.5

**Table 2:** Synergistic activity of subinhibitory concentrations of *Humulus lupulus* constituents and antibiotics belonging to various therapeutic classes, determined by the E-test strip/agar dilution method against *S. aureus* ATCC 29213.

Treatment	MIC [*µ*g/mL]		
	Oxacillin	Vancomycin	Linezolid
(Control)	0.125	1.0	0.5
Hop cones extract			
1/2 MIC (15.6 *µ*g/mL)	0.094	1.0	0.38
1/4 MIC (7.8 *µ*g/mL)	0.094	1.0	0.5
Spent hops extract			
1/2 MIC (1000 *µ*g/mL)	0.094	1.0	0.5
1/4 MIC (500 *µ*g/mL)	0.125	1.0	n.t.
Xanthohumol			
1/2 MIC (7.8 *µ*g/mL)	0.064	1.0	0.38
1/4 MIC (3.9 *µ*g/mL)	0.094	1.0	0.5

n.t.: not tested.
